# Prognostic Implication of Energy Metabolism-Related Gene Signatures in Lung Adenocarcinoma

**DOI:** 10.3389/fonc.2022.867470

**Published:** 2022-04-14

**Authors:** Teng Mu, Haoran Li, Xiangnan Li

**Affiliations:** ^1^ Department of Thoracic Surgery, The First Affiliated Hospital of Zhengzhou University, Zhengzhou, China; ^2^ Department of Thoracic Surgery, Peking University People’s Hospital, Beijing, China

**Keywords:** lung adenocarcinoma, energy metabolism, risk model, prognosis, nomogram

## Abstract

**Background:**

Lung adenocarcinoma (LUAD) is the major non-small-cell lung cancer pathological subtype with poor prognosis worldwide. Herein, we aimed to build an energy metabolism-associated prognostic gene signature to predict patient survival.

**Methods:**

The gene expression profiles of patients with LUAD were downloaded from the TCGA and GEO databases, and energy metabolism (EM)-related genes were downloaded from the GeneCards database. Univariate Cox and LASSO analyses were performed to identify the prognostic EM-associated gene signatures. Kaplan–Meier and receiver operating characteristic (ROC) curves were plotted to validate the predictive effect of the prognostic signatures. A CIBERSORT analysis was used to evaluate the correlation between the risk model and immune cells. A nomogram was used to predict the survival probability of LUAD based on a risk model.

**Results:**

We constructed a prognostic signature comprising 13 EM-related genes (AGER, AHSG, ALDH2, CIDEC, CYP17A1, FBP1, GNB3, GZMB, IGFBP1, SORD, SOX2, TRH and TYMS). The Kaplan–Meier curves validated the good predictive ability of the prognostic signature in TCGA AND two GEO datasets (p<0.0001, p=0.00021, and p=0.0034, respectively). The area under the curve (AUC) of the ROC curves also validated the predictive accuracy of the risk model. We built a nomogram to predict the survival probability of LUAD, and the calibration curves showed good predictive ability. Finally, a functional analysis also unveiled the different immune statuses between the two different risk groups.

**Conclusion:**

Our study constructed and verified a novel EM-related prognostic gene signature that could improve the individualized prediction of survival probability in LUAD.

## Introduction

Lung adenocarcinoma (LUAD) is the major lung cancer pathological subtype, and the 5-year survival rate remains very poor (1). The high mortality of lung adenocarcinoma is mostly due to the presence of metastatic lesions when diagnosed (2). Although treatment has embraced substantial advances over the past decade, complete surgical resection is still the most effective therapy. Therefore, novel biomarkers to predict the prognosis of patients with LUAD are urgently needed.

Cancer energy metabolism enabling tumor cells to produce adenosine triphosphate to maintain the reduction–oxidation balance and vital macromolecular biosynthesis for cell growth, migration and invasion has long been a hallmark of cancer cells (3). The Warburg phenomenon was found to be the first tumor energy metabolism alteration, and it comprises an increase in glycolysis that is maintained under conditions of high oxygen concentration (4). Glucose in cancer cells is the main source of energy, and cancer cells are usually programmed to increase glucose intake. Moreover, cancer cells prefer the nonoxidative metabolism of glucose, which promotes proliferation, growth and migration (5). Therefore, a deeper understanding of the relationship between energy metabolism and cancer cells might provide novel target therapies. In recent years, many studies have provided evidence for treating or diagnosing lung cancer (6–10). For instance, lnc-IGFBP4–1 is significantly upregulated in lung cancer tissues and plays a positive role in cell proliferation and metastasis through a potential mechanism of reprogramming tumor cell energy metabolism, and it may be a promising biomarker as a therapeutic target for lung cancer intervention (6).

In this study, energy metabolism-associated genes were collected. Gene expression profiles and the relative clinical information were downloaded from The Cancer Genome Atlas (TCGA) and Gene Expression Omnibus (GEO) databases. A 13-gene signature was found to build a prognostic risk model after differential expression and LASSO–Cox analysis. The risk model built *via* the TCGA dataset was validated by GEO external validation. Moreover, the risk model can be used as an independent prognostic factor for LUAD patients. The differences in critical biological function and immune cell distributions were also assessed. Finally, a nomogram was built to predict individual survival probability by integrating clinical information and the prognostic gene signature of patients.

## Methods

### Data Collection and Preprocessing

RNA-seq expression and clinical data, including 59 normal and 535 LUAD samples, were obtained from the TCGA database (https://portal.gdc.cancer.gov/) for analysis. Gene expression was normalized by the “limma” package in R. GSE31210 and GSE68465 were downloaded from the GEO database (http://ncbi.nlm.nih.gov/geo/) as validation sets consisting of 266 and 462 samples, respectively. Moreover, the relative clinical information of these samples was downloaded from GEO. Then, 1702 energy metabolism (EM)-related genes were obtained from the GeneCards database (https://www.genecards.org/). A total of 479 LUAD patients in the TCGA dataset with intact clinical information and 226 and 349 LUAD patients in GSE31210 and GSE68465, respectively, were finally enrolled in further study. The detailed characteristics of these patients are summarized in **Supplementary Table 1**, and the workflow of this study is shown in **Supplementary Figure 1**.

### Construction and Validation of the Prognosis EM-Related Gene Signature

Differentially expressed genes (DEGs) were identified by the “edgeR” package based on R software in the TCGA cohort. Then, a univariate Cox analysis of overall survival (OS) was performed to screen EM-related genes with potential prognostic value. p<0.05 was considered further. Next, a least absolute shrinkage and selection operator (LASSO) regression model was built to determine the optimal value of λ and construct a prognostic gene signature. The LASSO algorithm was used for variable selection and shrinkage based on the “glmnet” R package. After that, the risk scores of the included patients were calculated according to the gene expression level. Additionally, receiver operating characteristic (ROC) curves and Kaplan–Meier plots were plotted. To validate gene signature model robustness, the risk scores were also calculated in the GEO dataset (GSE31210 and GSE68465). ROC curves were used to analyze the prognostic value in validation sets. Moreover, survival analyses were performed in GEO datasets in R with the “survival” package. In addition, genetic alterations of survival-associated EM-related genes were assessed using cBioPortal (http://www.cbioportal.org/) for Cancer Genomics.

### Gene Expression and Kaplan–Meier Plotter

The expression of gene candidates was explored in The Human Protein Atlas (https://www.proteinatlas.org/), and the KM plot was obtained in Kaplan–Meier Plotter (http://www.kmplot.com/).

### Development of a Nomogram and Evaluation of Immune Cell Distribution

Sex, smoking status, age and risk level were used to construct a nomogram based on the “survival” and “rms” packages in R. Then, calibration curves were plotted to evaluate the effectiveness of the nomogram in the GEO validation sets. CIBERSORT (11) (https://cibersort.stanford.edu/) was used to estimate the differences in the high-risk vs. low-risk score groups using the Sangerbox tool (http://www.sangerbox.com/).

### Statistical Analysis

All statistical analyses were conducted based on SPSS (version 22.0) or R version 4.0.3 software. Differences in proportions were compared by a chi-squared test. Patients were assigned to the high-risk or low-risk groups according to the risk score. A Kaplan–Meier analysis with the log-rank test was used to evaluate the OS between these two groups. A Cox hazard regression model analysis was conducted to identify independent prognostic factors. p<0.05 was considered significant.

## Results

### Identification of Prognostic EM-Related DEGs in the TCGA Dataset

**Figure 1:**
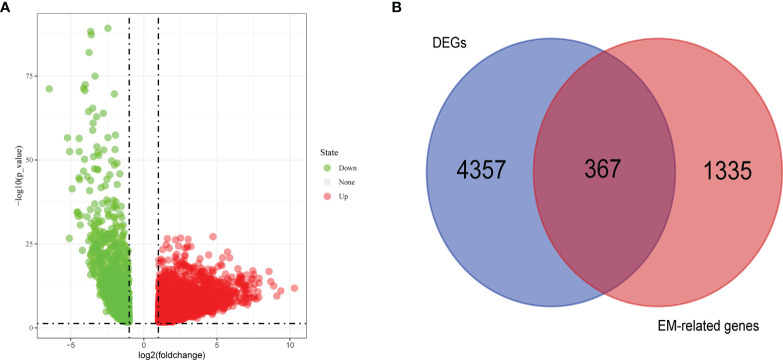
Volcano plot of differentially expressed genes (DEGs) in TCGA cohort **(A)** and Venn chart showed the number of DEGs associated with energy metabolism related genes **(B)**.

First, we found the DEGs in the TCGA data (**Figure 1A**) by comparing the gene expression levels in tumor and normal tissues (p<0.05) and searched the genes associated with energy metabolism in the GeneCards database. A total of 4724 DEGs were found, 1602 of which were downregulated and 3122 of which were upregulated. A total of 1702 EM-associated genes with relevance scores >7 were chosen to generate prognostic gene signatures. Three hundred and sixty-seven EM-related genes were subjected to further analysis (**Figure 1B**). These genes were all chosen for the univariate Cox regression analysis, and we found that 16 genes were significantly associated with OS in TCGA lung adenocarcinoma (LUAD).

### Construction of Prognostic Signature for TCGA LUAD

**Figure 2:**
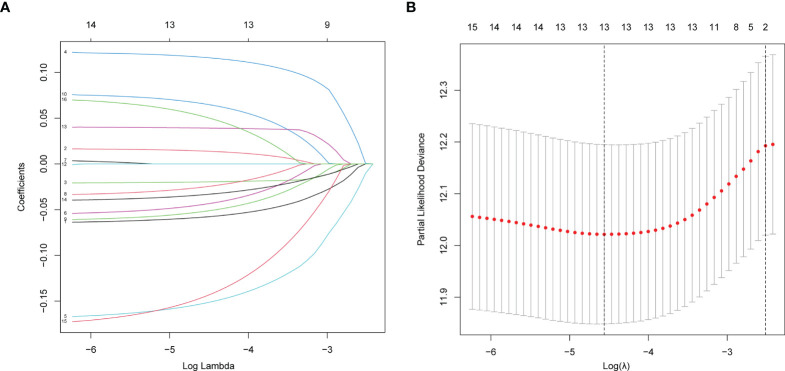
Establishment of prognostic gene signature by LASSO regression analysis. **(A)** LASSO coefficient profiles of the 16 genes in LUAD. A coefficient profile plot was generated against the log (lambda) sequence. **(B)** Selection of the optimal parameter (lambda) in the LASSO model for LUAD.

A LASSO regression analysis was applied to establish a prognostic gene signature using the 16 genes mentioned above (**Figures 2A, B**). A 13-gene signature involving AGER, AHSG, ALDH2, CIDEC, CYP17A1, FBP1, GNB3, GZMB, IGFBP1, SORD, SOX2, TRH and TYMS was identified by the optimal value of λ. Information on these genes is summarized in **Supplementary Table 2**.

A survival analysis was performed using the Kaplan–Meier plotter database, and we searched the expression of proteins encoded by these genes in The Human Protein Atlas (**Supplementary Figures 2**, **3**). The risk scores were also calculated and applied to predict prognosis with the median risk scores as the cutoff value to separate patients into a low-risk group and high-risk group.

**Figure 3:**
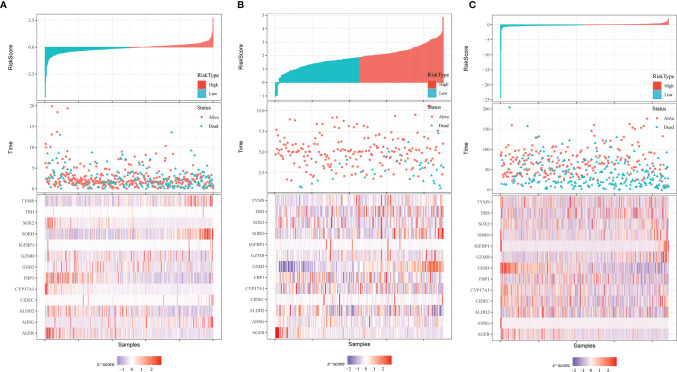
Distribution of risk score, OS, survival status (red dots indicate alive, blue dots indicate death) and the thirteen genes expression heatmaps in TCGA data **(A)**, GSE31210 **(B)** and GSE68465 **(C)**.

**Figure 4:**
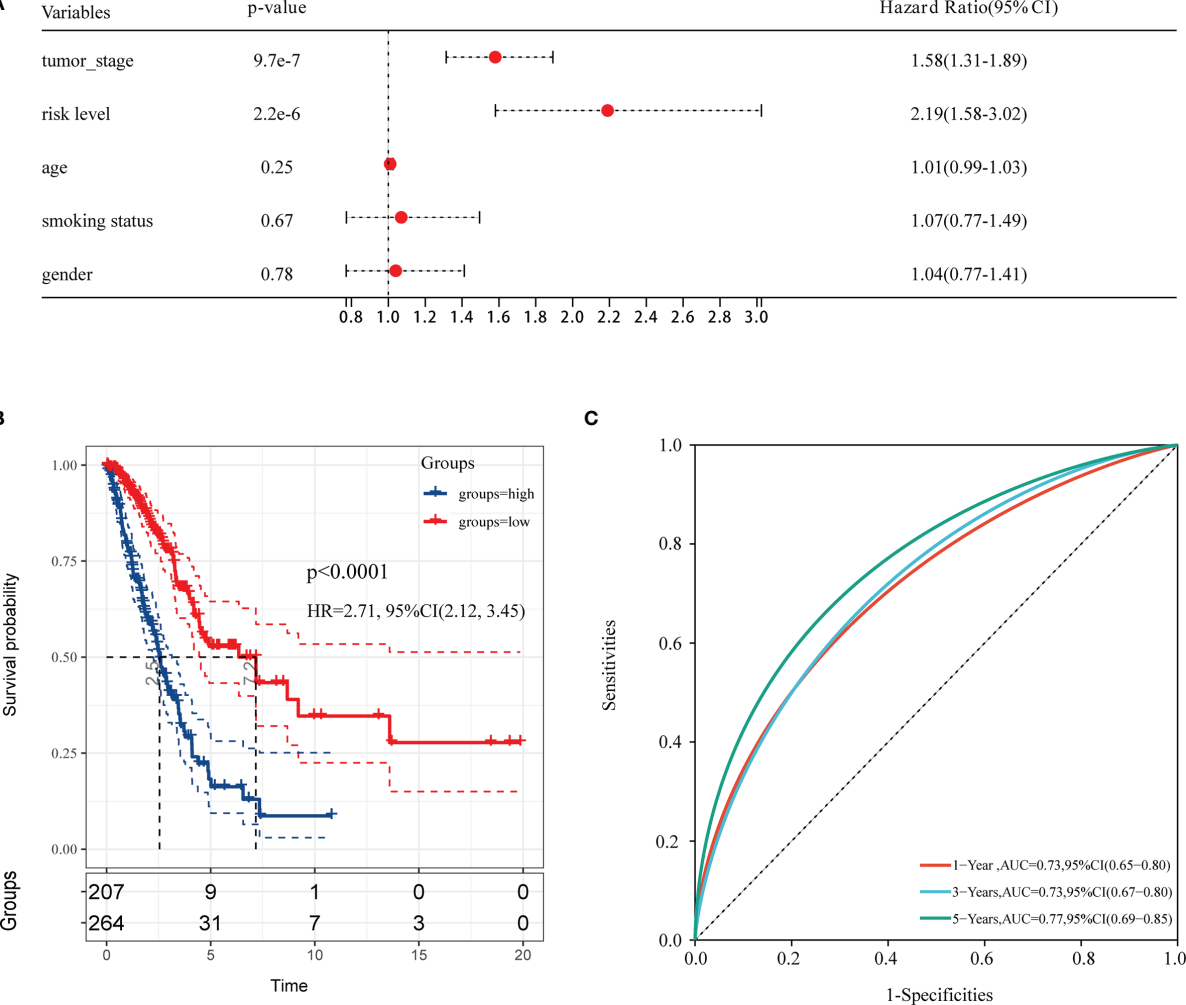
The forest plot of multivariable Cox analysis and evaluation of the performance of the risk model in the TCGA cohort. **(A)** The forest plot of association between risk factors and OS in LUAD. **(B)** The Kaplan–Meier (KM) curve of OS in the TCGA cohort. **(C)** ROC curves and area under the curve (AUC) for 1-, 3-, and 5-year survival in the TCGA cohort of the risk model.

A heatmap was plotted to assess gene expression in the high-risk and low-risk groups (**Figure 3A**). The distributions of the risk score of LUAD and the relation between the risk score and survival time are presented in **Figure 3A**. Next, a multivariate Cox analysis was performed. Tumor stage and the risk scores were significantly associated with OS in LUAD patients (**Figure 4A**). A KM plot was also constructed, and we found that patients with high risk scores survived significantly shorter than those with low risk scores (**Figure 4B**). The predictive value of the risk score for OS was assessed by ROC curves, and the AUCs were 0.73 at 1 year, 0.73 at 3 years and 0.77 at 5 years (**Figure 4C**).

### Validation of the EM-Associated Gene Signature in GEO Datasets

**Figure 5:**
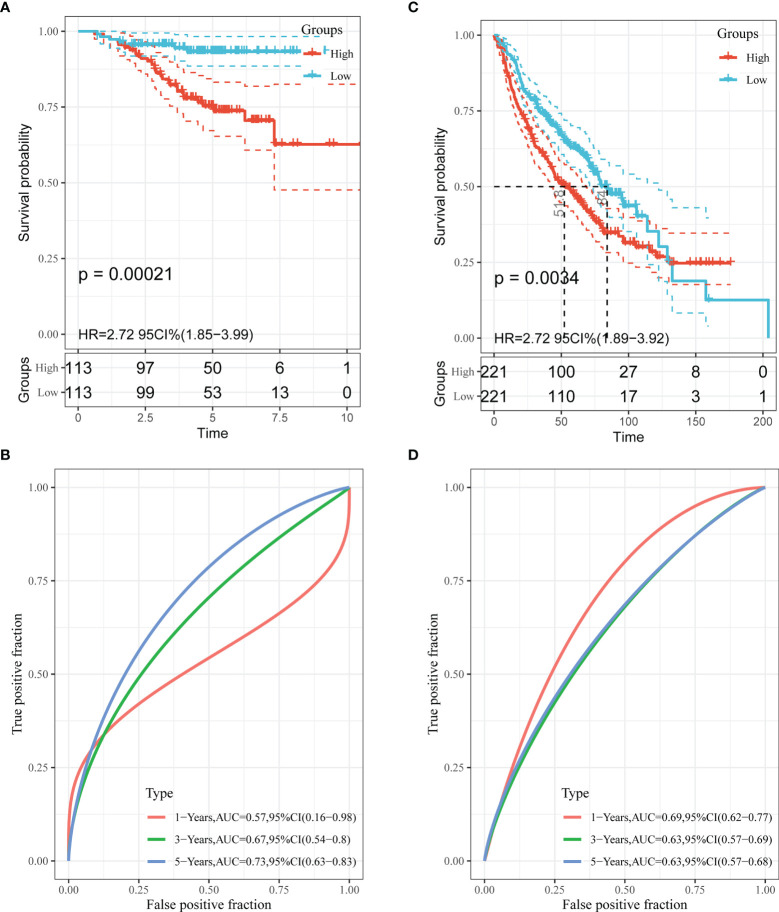
Validation of the performance of the risk model in the GEO cohorts. Kaplan–Meier curves of the OS in the GSE31210 cohort **(A)** and GSE68465 cohort **(C)**. ROC curves and area under the curve (AUC) for 1-, 3-, and 5-year survival in the GSE31210 cohort **(B)** and GSE68465 cohort **(D)**.

To test the effectiveness of the model built from the TCGA data, the patients from the GSE31210 and GSE68465 datasets were also divided into high- and low-risk groups using a similar formula to TCGA data. Heatmaps showed EM gene expression in the two groups, and the relationship between risk scores and survival time is also plotted in **Figures 3B, C**. Similar to the TCGA data, the survival analyses found that patients with higher risk scores had poorer OS (p=0.00021 and p=0.0034) (**Figures 5A, C**). In addition, we plotted the ROC curves to evaluate the robustness of the gene signature model. In the GSE31210 dataset, the AUC was 0.57 at one year, 0.67 at three years and 0.73 at five years (**Figure 5B**). In the GSE68465 dataset, the AUCs were 0.69, 0.63 and 0.63 at one year, three years and five years, respectively (**Figure 5D**).

### Construction and Validation of a Prognostic Nomogram

**Figure 6:**
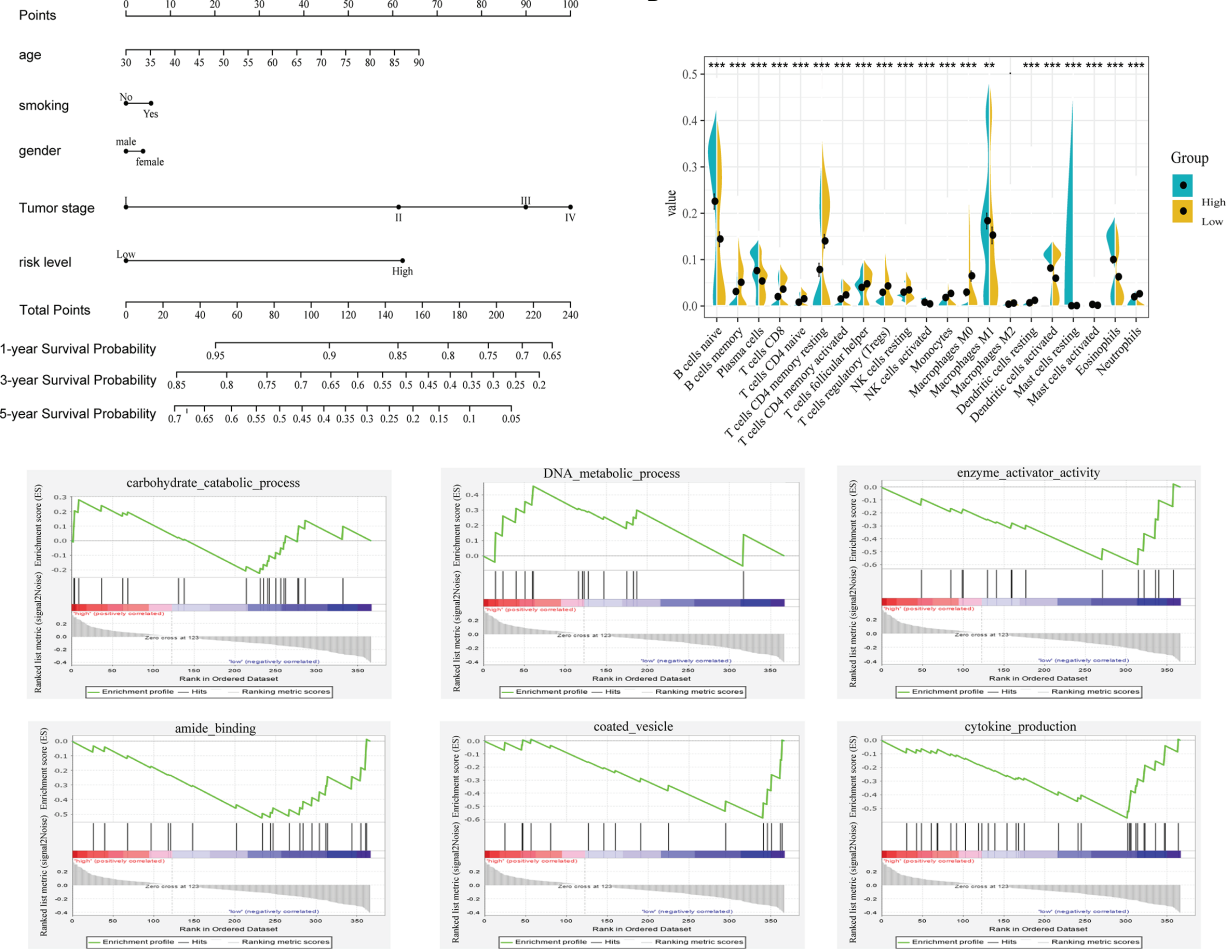
**(A)** Nomogram predicting the OS in LUAD patients containing the risk level. **(B)** 22 immune cell distributions in LUAD based on risk level using CIBERSORT. (., NS; **, p<0.01; ***, p<0.001) **(C)** GSEA analysis based on the median value of risk score.

A prognostic nomogram can be applied to evaluate an individual’s risk in the clinical setting by integrating several risks as an effective tool (12). Age, gender, smoking status, tumor stage and risk level were the parameters included in the nomogram (**Figure 6A**). The calibration curves in the GEO datasets demonstrated that the actual and predicted survival matched well (**Supplementary Figure 4**). For instance, a 65-year (38 points) male (0 points) LUAD patient who smoked (5 points) had high risk scores (62 points) and his tumor was stage III (90 points) would obtain 195 points. His 1-year, 3-year and 5-year survival rates would be 77%, 34% and 9%, respectively.

### Functional Analysis in TCGA Based on the Risk Score

To unveil the differences in biological function between the high- and low-risk groups, we performed gene set enrichment analysis (GSEA) using the GSEA online analysis tool (www.gsea-msigdb.org/). As shown in **Figure 6C**, carbohydrate catabolic process, DNA metabolic process, enzyme activator activity, amide binding, coated vesicle and cytokine production were showed differently in these two groups. Then, we investigated the relationship between immune cells and the risk group using CIBERSORT (**Figure 6B**). The distributions of most immune cells in the two groups were diverse, especially CD8+ T cells, which was consistent with the GSEA results (regulation of the immune system process).

## Discussion

The prognosis of LUAD is usually poor because of the late diagnosis and the limitations of current therapies. Complete surgical resection is only a robust therapy; however, patients with LUAD often lose the opportunity to undergo surgery because the neoplasm is already in advanced stages at diagnosis. Therefore, novel biomarkers need to be found in the prediction of the survival probability of LUAD.

Reprogrammed energy metabolism, such as aerobic glycolysis, is considered a hallmark of cancer (13). LDH-A, a metabolic enzyme that converts pyruvate to lactate, was identified as the first target of the MYC oncogene, and MYC-driven tumors in a xenograft model were diminished by targeting the LDH-A gene (13). Oncogenic activation also promotes mitochondrial metabolism to generate ATP and TCA cycle intermediates for macromolecule synthesis; for example, citrate is the precursor for lipid or nucleotide synthesis (14). Thus, considering EM-related genes as the target therapy is promising. We are particularly interested in the exploration of the relationship between the prognosis of LUAD and EM-related genes and want to find some gene signatures from these genes to be a panel of prognostic markers.

In our study, public gene expression data from the TCGA and GEO databases were used to construct a 13-gene signature for a prognostic risk model after univariate Cox and LASSO regression analyses. The risk model comprising *AGER, AHSG, ALDH2, CIDEC, CYP17A1, FBP1, GNB3, GZMB, IGFBP1, SORD, SOX2, TRH* and *TYMS* was effective and stable in predicting patient prognosis after validation of GEO data. Moreover, a nomogram was built to predict the survival probability. The GSEA and immune cell analysis demonstrated that the two groups divided by risk level were significantly different in enzyme activation, immune system development, cell differentiation, cytokine production and regulation of immune system processes. In summary, we found an effective panel of 13 gene signatures for predicting prognosis and a nomogram to assess the survival probability of LUAD patients.

The role of AGER in tumorigenesis remains controversial. Wang et al. showed that *AGER* overexpression in H1299 cells displayed decreased cell viability, proliferation, migration and invasion abilities and significantly increased levels of apoptosis compared with control cells (15), and their findings were consistent with those of Zhang (16). However, AGER is upregulated in cervical cancer, promoting proliferation and migration of cervical squamous cancer cells (17). *AHSG* and *IGFBP2* levels were increased in lung patients with malignant pleural effusion but those with nonmalignant pleural effusion, and the authors simultaneously demonstrated the extracellular function of *IGFBP2* in migration in lung cancer cells (18). Accumulating evidence suggests that *ALDH2* dysfuction contributes to human diseases such as cancers, and *ALDH2* is suppressed in human lung adenocarcinoma (19). Additionally, Guo et al. illustrated that ARF-like GTPase 14 plays an important role in the pathogenesis of LUAD through the CIDEC/ERK/p38 signaling pathway (20).

*CYP17A1*, which converts testosterone to estradiol, is a promising non-small lung cancer (NSCLC) susceptible candidate gene, but its polymorphisms are not associated with NSCLC development in Asian populations (21). A recent study found that aberrant expression of *FBP1* in natural killer cells elicited their dysfunction by inhibiting glycolysis and impairing viability (22). In addition, a germline variation of GZMB and low baseline serum level of granzyme B were associated with worse clinical outcome in NSCLC (23). Schaal et al. demonstrated SOX2 to be indispensable for self-renewal and stemness in NSCLC cells (24). *GNB3, TRH*, and *TYMS* also play important roles in human cancers (25–27). For instance, the GNB3 825C>T polymorphism might influence development of metastasis in low-grade breast tumors (25).

In addition, we found different distributions of immune cells in the high- and low-risk groups. CD8+T cells are often considered the main effector cells of antitumor immunity, and we found a high distribution in the low-risk group. Thus, the immunological function of LUAD patients with high risk levels may be compromised, and further validation is needed.

The advantage of our study is that we identified a prognostic model with a 13-gene signature that predicts one-, three-, five-year survival with relatively high AUCs in both the TCGA and GEO databases. In addition, we established a nomogram to predict survival probability, and its calibration curve also showed relatively high accuracy. However, our study has limitations. Our results are based on a bioinformation analysis without experimental validation, and the functions of these 13 genes in LUAD need to be further studied.

In summary, we offer some new understandings on the association between EM and LUAD. We explored EM-related gene expression and its prognostic implication in LUAD and identified an EM-associated gene signature to establish a risk model with good performance of prognostic prediction. Simultaneously, we built a nomogram to predict the survival probabilities of LUAD patients, and the calibration curves also showed good predictive ability.

## Data Availability Statement

The datasets presented in this study can be found in online repositories. The names of the repository/repositories and accession number(s) can be found in the article/**Supplementary Material**.

## Author Contributions

XL designed the overall study. TM and HL performed the data collection and analysis. TM, HL, and XL wrote and revised the manuscript. All authors contributed to the article and approved the submitted version.

## Conflict of Interest

The authors declare that the research was conducted in the absence of any commercial or financial relationships that could be construed as a potential conflict of interest.

## Publisher’s Note

All claims expressed in this article are solely those of the authors and do not necessarily represent those of their affiliated organizations, or those of the publisher, the editors and the reviewers. Any product that may be evaluated in this article, or claim that may be made by its manufacturer, is not guaranteed or endorsed by the publisher.

## Supplementary Material

Supplementary Figure 1 Flowchart presenting the process of establishing the gene signature and prognostic nomogram of LUAD in this study.

Supplementary Figure 2 The expression of 13 genes in six Human Protein atlas and Kaplan–Meier curves of the OS of CYP17A1.

Supplementary Figure 3 Kaplan–Meier curves of the OS of 12 genes in the Kaplan-Meier plotter database.

Supplementary Figure 4 Calibration curves of 1-, 3-, and 5-year OS in GSE31210 cohort **(A–C)** and GSE68465 cohort **(D–F)**. The Y axis represents the actual OS while the X axis represents nomogram predicted OS.

## Abbreviations

LUAD, lung adenocarcinoma; EM, energy metabolism; ROC, receiver operating characteristic; AUC, area under the curve; NSCLC, non-small cell lung cancer; DEGs, differentially expressed genes; OS, overall survival; LASSO, least absolute shrinkage and selection operator; GSEA, gene set enrichment analysis.
