# Supplementary figures and images for: Prognostic Implication of Energy Metabolism-Related Gene Signatures in Lung Adenocarcinoma
Source: Front Oncol. 2022 Apr 14;12:867470. doi: 10.3389/fonc.2022.867470 (PMC9047773; doi:10.3389/fonc.2022.867470)

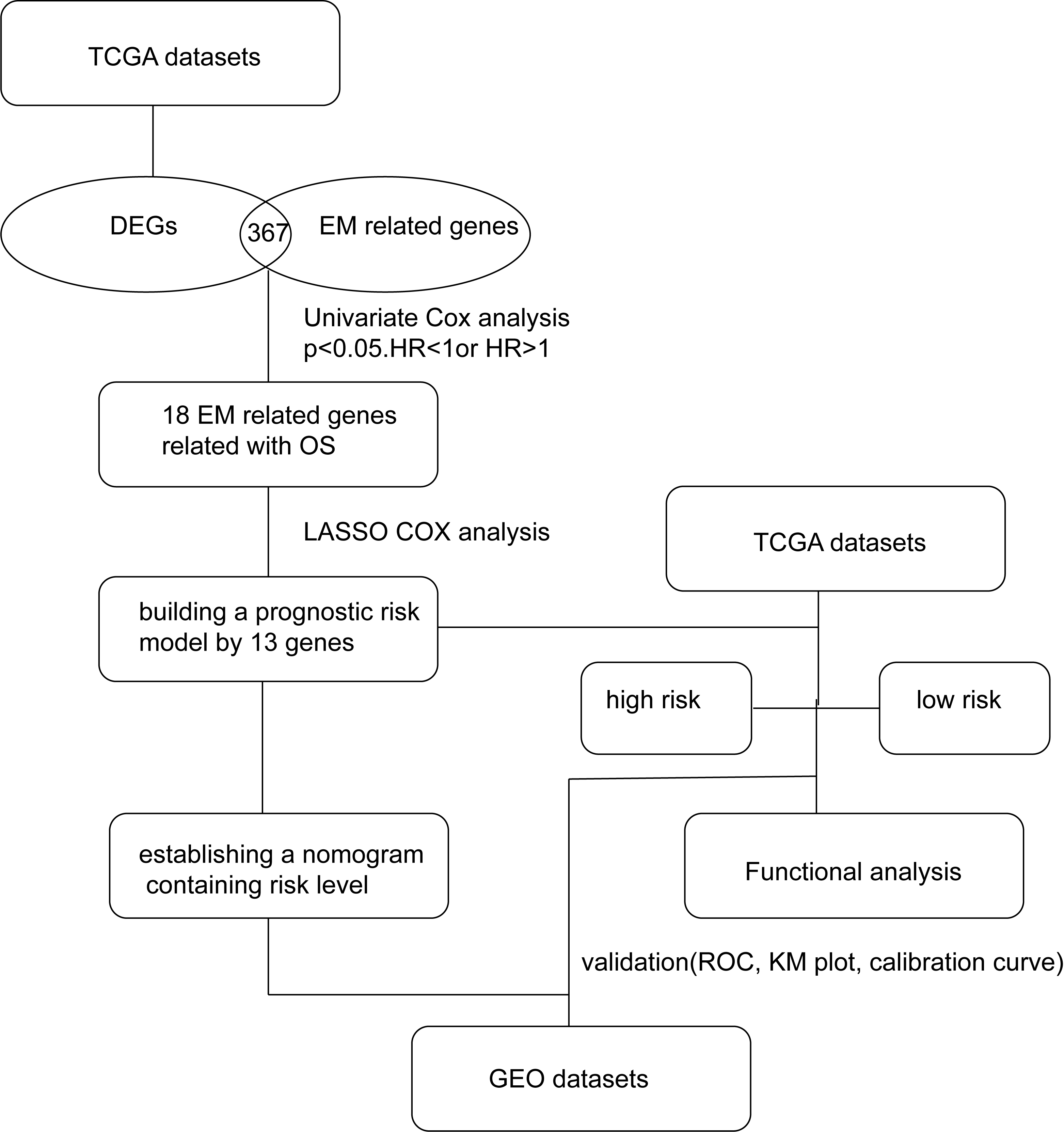

Supplement: Supplementary Figure 1 — Flowchart presenting the process of establishing the gene signature and prognostic nomogram of LUAD in this study. [file Image_1.tif]

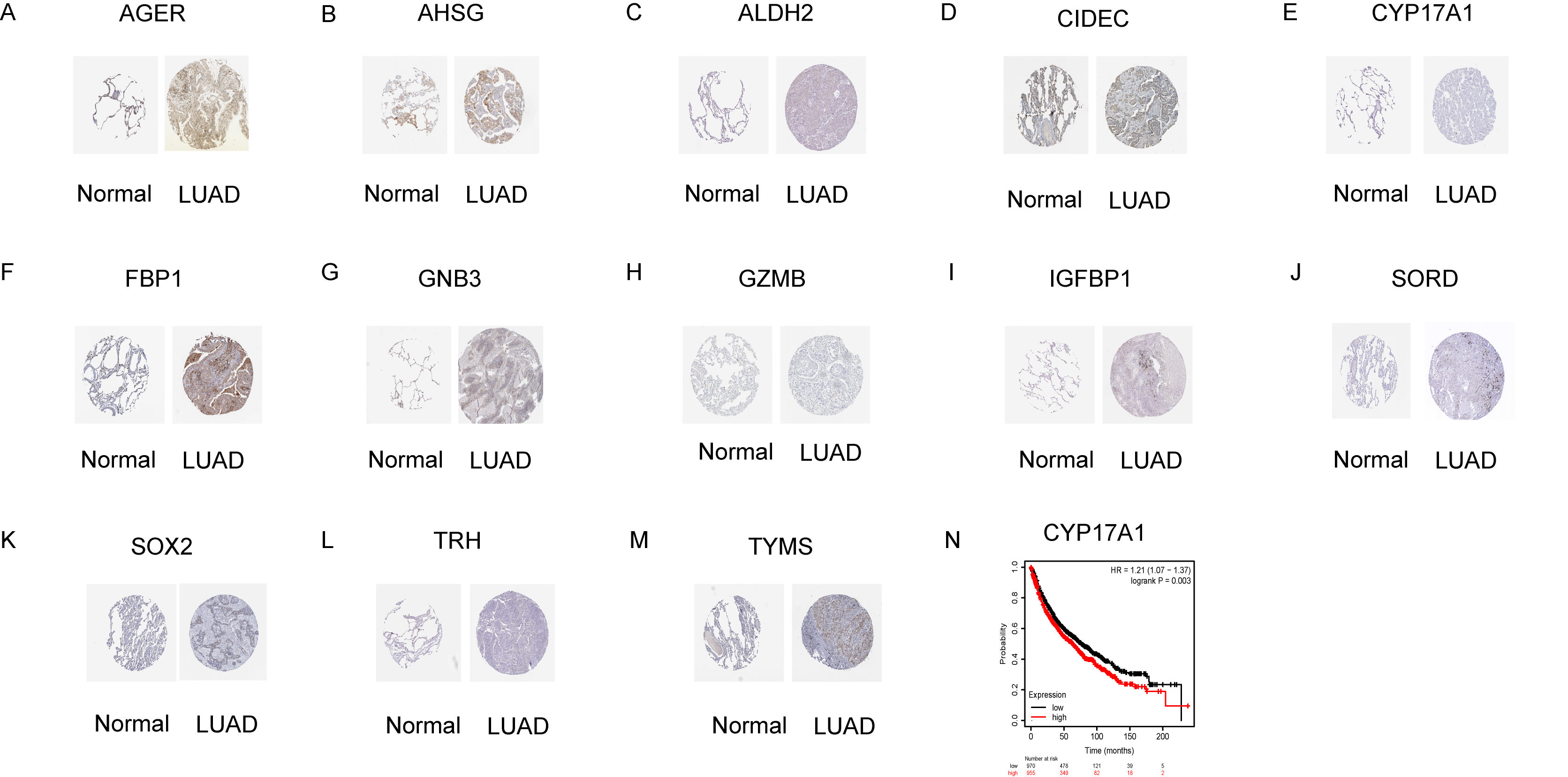

Supplement: Supplementary Figure 2 — The expression of 13 genes in six Human Protein atlas and Kaplan–Meier curves of the OS of CYP17A1. [file Image_2.tif]

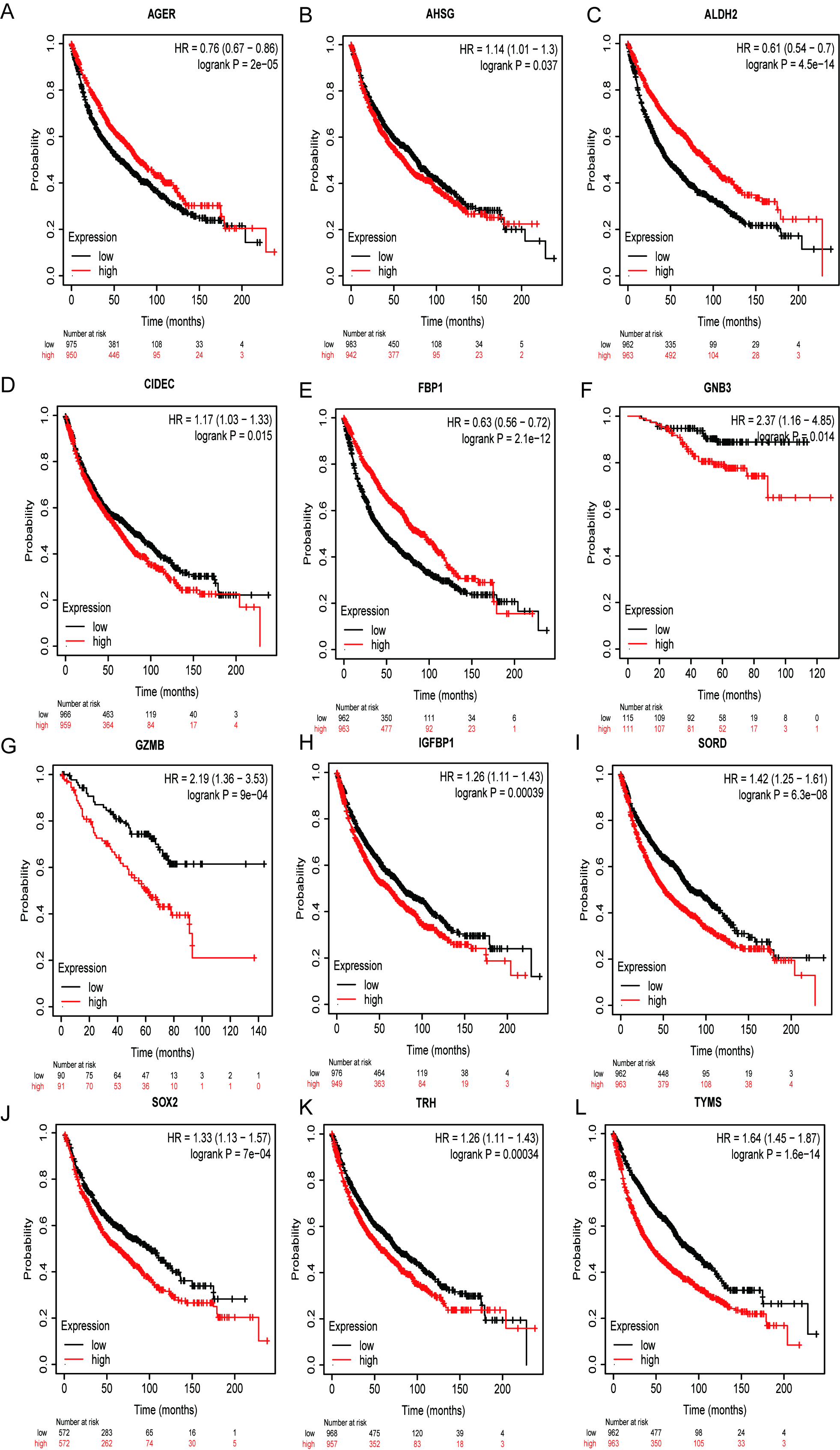

Supplement: Supplementary Figure 3 — Kaplan–Meier curves of the OS of 12 genes in the Kaplan-Meier plotter database. [file Image_3.tif]

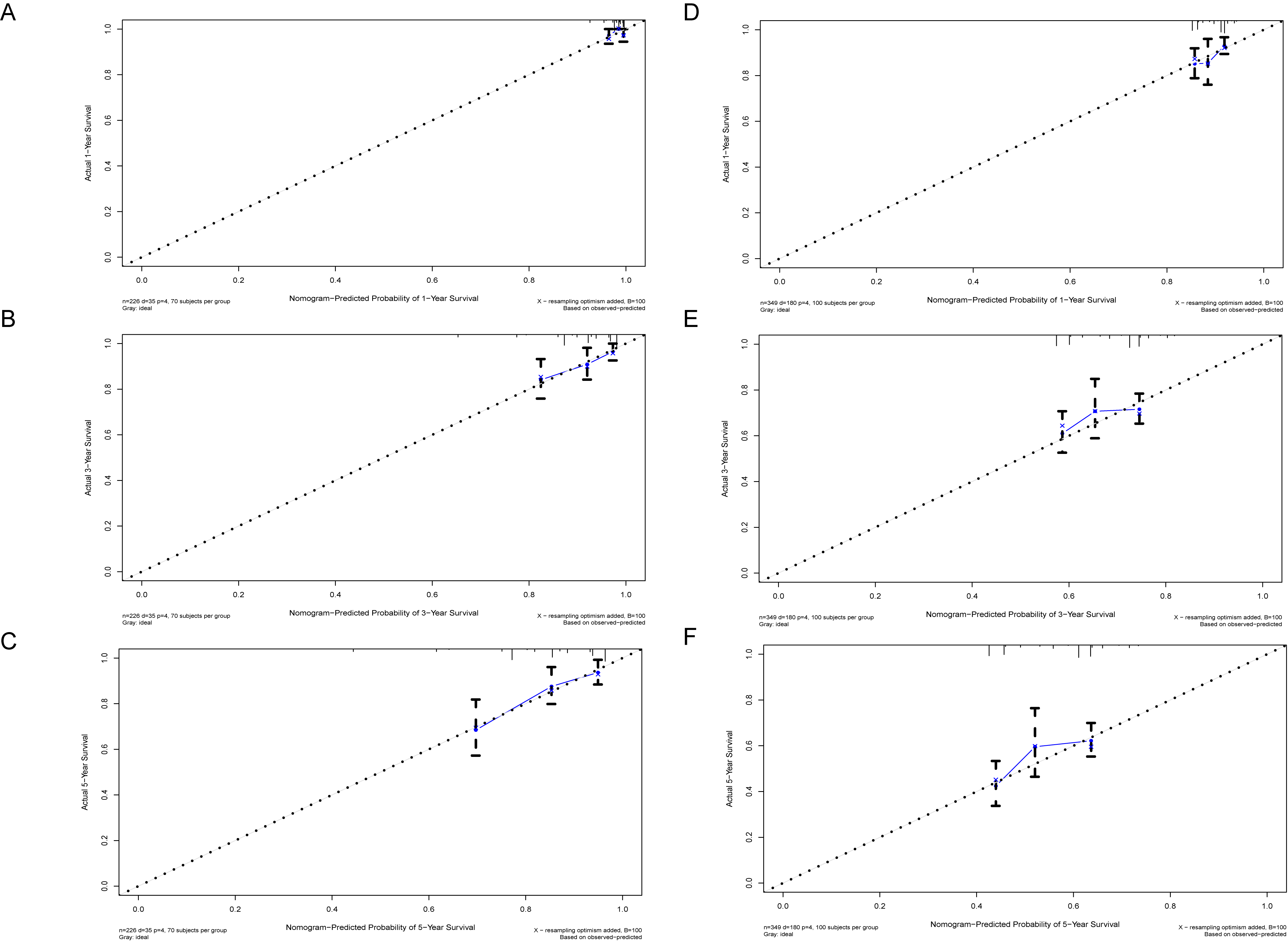

Supplement: Supplementary Figure 4 — Calibration curves of 1-, 3-, and 5-year OS in GSE31210 cohort (A–C) and GSE68465 cohort (D–F). The Y axis represents the actual OS while the X axis represents nomogram predicted OS. [file Image_4.tif]
