# Supplementary material for: Prognostic Implication of Energy Metabolism-Related Gene Signatures in Lung Adenocarcinoma
Source: Front Oncol. 2022 Apr 14;12:867470. doi: 10.3389/fonc.2022.867470 (PMC9047773; doi:10.3389/fonc.2022.867470)
Supplement: Supplementary file 5 [file Table_1.docx]

Supplement table1. clinical characteristics of LUAD patients included in this study.

|  | TCGA | GSE31210 | GSE68465 |
| --- | --- | --- | --- |
| No. of patients | 479 | 226 | 349 |
| Age |  |  |  |
| <=65 | 225 | 176 | 186 |
| >65 | 254 | 50 | 163 |
| Gender |  |  |  |
| Male | 217 | 105 | 162 |
| Female | 262 | 121 | 187 |
| Smoking status |  |  |  |
| Yes | 334 | 111 | 300 |
| No | 145 | 115 | 49 |
| Survival status |  |  |  |
| Dead | 173 | 35 | 180 |
| Alive | 306 | 191 | 169 |
