# Supplementary material for: Prognostic Implication of Energy Metabolism-Related Gene Signatures in Lung Adenocarcinoma
Source: Front Oncol. 2022 Apr 14;12:867470. doi: 10.3389/fonc.2022.867470 (PMC9047773; doi:10.3389/fonc.2022.867470)
Supplement: Supplementary file 6 [file Table_2.docx]

Supplement table 2. The information of prognostic gene signatures.

| No. | Gene symbol | Full name | Pivotal metabolic pathway |
| --- | --- | --- | --- |
| 1 | AGER | Advanced Glycosylation End-Product Specific Receptor | Lipid metabolism |
| 2 | AHSG | Alpha 2-HS Glycoprotein | Protein families: metabolism |
| 3 | ALDH2 | Aldehyde Dehydrogenase 2 Family Member | Citrate cycle |
| 4 | CIDEC | Cell Death Inducing DFFA Like Effector C | Lipid metabolism |
| 5 | CYP17A1 | Cytochrome P450 Family 17 Subfamily A Member 1 | Carbohydrate metabolism |
| 6 | FBP1 | Fructose-Bisphosphatase 1 | Glycolysis / Gluconeogenesis |
| 7 | GNB3 | G Protein Subunit Beta 3 | Nucleotide metabolism |
| 8 | GZMB | Granzyme B | Protein metabolism |
| 9 | IGFBP1 | Insulin Like Growth Factor Binding Protein 1 | Protein metabolism |
| 10 | SORD | Sorbitol Dehydrogenase | Fructose and mannose metabolism |
| 11 | SOX2 | SRY-Box Transcription Factor 2 | Hormone metabolism |
| 12 | TRH | Thyrotropin Releasing Hormone | Hormone metabolism |
| 13 | TYMS | Thymidylate Synthetase | Pyrimidine metabolism |
